# Neuroimaging Markers for Studying Gulf-War Illness: Single-Subject Level Analytical Method Based on Machine Learning

**DOI:** 10.3390/brainsci10110884

**Published:** 2020-11-20

**Authors:** Yi Guan, Chia-Hsin Cheng, Weifan Chen, Yingqi Zhang, Sophia Koo, Maxine Krengel, Patricia Janulewicz, Rosemary Toomey, Ehwa Yang, Rafeeque Bhadelia, Lea Steele, Jae-Hun Kim, Kimberly Sullivan, Bang-Bon Koo

**Affiliations:** 1 School of Medicine, Boston University, Boston, MA 02118, USA; guanyi1@bu.edu (Y.G.); chiahsin@bu.edu (C.-H.C.); wfchen@bu.edu (W.C.); yqz2019@bu.edu (Y.Z.); sskoo@bu.edu (S.K.); mhk@bu.edu (M.K.); toomey@bu.edu (R.T.); 2 School of Public Health, Boston University, Boston, MA 02118, USA; paj@bu.edu; 3 Department of Radiology, Samsung Medical Center, Sungkyunkwan University School of Medicine, Seoul 06351, Korea; ehwayang@gmail.com (E.Y.); jaehun1115.kim@samsung.com (J.-H.K.); 4 Department of Radiology, Beth Israel Deaconess Medical Center, Harvard Medical School, Boston, MA 02115, USA; rbhadelia@gmail.com; 5 Neuropsychiatry Division, Department of Psychiatry and Behavioral Sciences, Baylor College of Medicine, Houston, TX 77030, USA; Lea.Steele@bcm.edu

**Keywords:** Gulf War illness, MRI, objective biomarker, machine learning, Kansas case criteria, diffusion, grey matter, neurite density imaging

## Abstract

Gulf War illness (GWI) refers to the multitude of chronic health symptoms, spanning from fatigue, musculoskeletal pain, and neurological complaints to respiratory, gastrointestinal, and dermatologic symptoms experienced by about 250,000 GW veterans who served in the 1991 Gulf War (GW). Longitudinal studies showed that the severity of these symptoms often remain unchanged even years after the GW, and these veterans with GWI continue to have poorer general health and increased chronic medical conditions than their non-deployed counterparts. For better management and treatment of this condition, there is an urgent need for developing objective biomarkers that can help with simple and accurate diagnosis of GWI. In this study, we applied multiple neuroimaging techniques, including T1-weighted magnetic resonance imaging (T1W-MRI), diffusion tensor imaging (DTI), and novel neurite density imaging (NDI) to perform both a group-level statistical comparison and a single-subject level machine learning (ML) analysis to identify diagnostic imaging features of GWI. Our results supported NDI as the most sensitive in defining GWI characteristics. In particular, our classifier trained with white matter NDI features achieved an accuracy of 90% and F-score of 0.941 for classifying GWI cases from controls after the cross-validation. These results are consistent with our previous study which suggests that NDI measures are sensitive to the microstructural and macrostructural changes in the brain of veterans with GWI, which can be valuable for designing better diagnosis method and treatment efficacy studies.

## 1. Introduction

Gulf War illness (GWI) refers to the variety of chronic symptoms experienced by about 250,000 United States veterans who served in the 1991 Gulf War (GW) [1]. According to the Kansas case criteria, symptoms of GWI fall into six categories: fatigue (fatigue and sleep problems), pain (joint and muscle), neurological (cognitive, mood, headache, and dizziness), respiratory (persistent cough and wheezing), gastrointestinal (diarrhea and nausea), and skin (rashes and other) problems. Exposure to neurotoxicant chemicals (organophosphate pesticides and sarin) during the war and other central nervous system (CNS) damage, such as mild traumatic brain injury (mTBI), are thought to have caused an innate immune over-response in the CNS, resulting in the development of these chronic GWI symptoms [2,3,4,5,6,7]). In order to meet the Kansas criteria for GWI, veterans must display chronic symptoms in at least three of the six categories, without presenting concurrent psychiatric and medical disorders [8]. However, accurate diagnoses of GWI remained challenging due to the heterogeneous clinical presentation of this condition, as well as the level of subjectivity associated with self-reported symptoms and neurotoxicant exposure history [8,9,10]. To improve management and treatment of GWI, there is an urgent need for defining sensitive and objective biomarkers of the disorder.

Previous neuroimaging studies demonstrated distinct changes within brains of veterans with GWI, which may underlie physiological symptoms. For example, T1W-MRI studies showed that GW veterans with exposure to the neurotoxicant chemical sarin exhibit reduced gray matter (GM) and white matter (WM) volumes, as well as reductions in hippocampal subfield volumes when compared to non-exposed veterans [11,12]. More recent studies using diffusion tensor imaging (DTI) have shown greater hippocampal mean diffusivity (MD) and increased axial diffusivity (AD) in the WM of sarin and cyclosarin exposed GW veterans, which are correlated to fatigue, pain, or hyperalgesia, and may serve as a potential biomarker for GWI [13,14,15]. We have previously applied a novel MRI diffusion processing method, neurite density imaging (NDI), on high-order diffusion MRI to demonstrate that the NDI measure scan successfully identify and validate different levels of neurological abnormalities in veterans with GWI from the Boston Gulf War Illness Consortium cohort [16].

ML algorithms have been applied to study a wide range of neurological disorders, including Alzheimer’s disease, Parkinson’s disease, and traumatic brain injury [17,18]. These studies have reported promising results for identifying diagnostic biomarkers [19,20]. The ML approach have strengths on exploiting features from different domains (i.e., neuropsychological, genetic and neuroimaging) and providing further insights on the potential interactions between different markers for classifying illness [21]. For the current study, we aimed to expand our previous work (on NDI) to cross-compare different types of neuroimaging markers (T1W-MRI, DTI and NDI) to determine whether these measures are useful for single subject-level classification of GWI cases vs. controls. Specifically, we incorporated the machine learning (ML) framework to search out key imaging features valuable for defining GWI. Computerized models were then trained based on the selected features and tested for classifying veterans with GWI.

## 2. Methods

### 2.1. Participants

In this study, we included brain imaging data of 119 GW veterans from Boston University Gulf War Illness Consortium (GWIC) (Table 1). GWIC is a multi-site study designed to identify the etiology and potential biomarkers of GWI. The inclusion criterion was deployment to the GW between August 1990 and July 1991. The exclusion criteria included having a diagnosis of chronic medical illnesses that could otherwise account for the symptoms experienced by GW veterans, including autoimmune, CNS, or major psychiatric disorders that could affect the brain and immune functions (e.g., epilepsy, stroke, severe head injury, etc.). Each participant completed an assessment protocol of health surveys, a neuropsychological test battery, brain imaging, and collection of blood and saliva samples [2]. In this study, we utilized brain imaging outcomes to study GWI. All participants provided written informed consent to participate in the study. This study was reviewed and approved by the Boston University institutional review board.

#### Gulf War Illness Criteria and Symptom Surveys

GWI case status was defined from the Kansas GWI case definition, which requires multiple or moderate-to-severe chronic symptoms in at least three of six statistically defined symptom domains: fatigue/sleep problems, somatic pain, neurological cognitive/mood symptoms, gastrointestinal symptoms, respiratory symptoms, and skin abnormalities [8]. GWIC participants not meeting Kansas GWI or exclusionary criteria were considered controls. Veterans were excluded from being considered GWI cases, for purposes of the research study, if they reported being diagnosed by a physician with medical or psychiatric conditions that would account for their symptoms or interfere with their ability to report their symptoms. GWIC subjects were administered a general demographic information and medical conditions questionnaire and the Kansas Gulf War and health questionnaire for assessing symptoms [8,10]. Additional validated health symptom surveys were completed by study participants and included the multidimensional fatigue inventory (MFI-20), McGill pain inventory and the Pittsburgh sleep quality index (PSQI) where higher scores suggested worse conditions [22,23,24].

### 2.2. Image Acquisition

All veterans were scanned on an Achieva 3T whole-body MRI scanner (Philips Healthcare, Best, The Netherlands) at the Center of Biomedical Imaging, Boston University school of Medicine. T1W-MRI were obtained using an MPRAGE sequence developed by the Alzheimer’s disease neuroimaging initiative (ADNI) (Repetition time (TR) = 6.8 ms, Echo time (TE) = 3.1 ms, flip angle = 9°, slice thickness = 1.2 mm, 170 slices, Field of view (FOV) = 250 mm, matrix = 256 × 256) (accessible from http://adni.loni.usc.edu/). Diffusion MRI data were obtained using 124 gradient directions utilizing parallel imaging on a 16-channel parallel head coil (70 slices, TR = 13,214 ms, TE = 55 ms, with a matrix size of 128 × 128 yielding a resolution of 2.0 × 2.0 × 2.0 mm^3^, no slice gap). Multi-shell diffusion encodings with b-values 1000, 2000 and 3000 s/mm^2^ were acquired with a single-shot echo planar imaging (EPI) sequence, and 6 b = 0 s/mm^2^ field maps were collected in addition to distortion corrections built into the scanner.

### 2.3. Image Processing and Anatomical Defining

Structural T1W-MRI scans were analyzed with the Freesurfer package (version 6.0) to generate anatomical regions of interest (ROI) for assessing GM morphometric measures, and to provide GM anatomical co-registration references for diffusion images [25]. A total of 78 ROIs defined in the average template space were co-registered to each subject’s cortical surface by applying nonlinear co-registration parameters. All results were visually inspected for artifacts or incomplete segmentation. Fractional anisotropy (FA), mean diffusivity (MD), axial diffusivity (AD), and radial diffusivity (RD) maps were created using tract-based spatial statistics (TBSS), part of FSL package that projects all subjects’ diffusion tensor imaging (DTI) data onto a mean tract skeleton [26]. A total of 20 major WM tracts were defined using the Johns Hopkins University (JHU) white-matter tractography atlas provided in the FSL package, the same template was also used for special normalization and linear co-registration of diffusion MRIs [27,28].

### 2.4. High-Order Diffusion Processing

Microstructural diffusion measures were reconstructed from multi-shell diffusion MRI images containing 3 b-value encodings using the NDI model [16]. Two parameters, neurite density (ND) index and orientation dispersion (OD) index were extracted from the NDI model. In brief, ND is a fraction of tissue composed of neurites which include axons and dendrites, and OD provides the spatial configuration of the neurite structures based on the composite pattern of intra- and extracellular diffusivity [29]. For WM NDI measures, all subjects’ NDI data were registered to a common space based on nonlinear transformation and projected to the WM tract skeleton. Next the major WM tract ROIs were then applied to the skeletonized WM NDI maps to extract ROI-wise NDI measures [26]. For the GM diffusivity assessment, diffusion modeling parameters were determined by voxel wise iterative parameter selection method. We used the maximum likelihood estimation of model fitting error to define the optimal intrinsic free diffusivity parameters [30]. The optimal parameters were used to reconstruct the GM NDI maps and then merged into the 78 GM ROIs to extract ROI-wise NDI measures [30,31].

### 2.5. T1-Weighted MRI Measures

From the Freesurfer cortical reconstruction process of T1W-MRI, we extracted six measures per subject, including cortical thickness, cortical surface area, cortical volume (cVolume), subcortical GM volume (scVolume), WM volume, curvature (curv). Specifically, cortical thickness, surface area, volume, and curvature are extracted from 62 ROIs based on Desikan–Killiany–Tourville (DKT) atlas, while subcortical ROIs are defined by Freesurfer built-in atlas [31,32].

### 2.6. Statistical Analysis

From the data processing steps, we generated in total 14 types of imaging measures: 4 NDI, 4 DTI, and 6 T1-weighted morphometric measures. For each type of imaging measure, we conducted statistical comparisons of GWI cases vs. controls using linear regression models adjusting for age and sex, and then corrected for multiple comparison using false discovery rate (FDR) [33]. We reported t-values and FDR-corrected *p*-values (FDR-*p*), significant features are defined as FDR-*p* < 0.05.

### 2.7. Machine Learning Classification

Imaging measures described in the previous sections are used as pre-defined features for training ML classification models. Age- and sex-related confounds were removed from the raw data before training the model. This step is achieved by estimating the effects of age and sex on imaging measures using a linear regression model that is similar to a method applied in an early study [19]. For building the classifier for each imaging measure we adapted a reinforcement learning algorithm with artificial bee colony algorithm for feature selection (BSO: bee swarm optimization), and the K nearest neighbors (KNN) algorithm for classification training and performance evaluation [34,35].

#### 2.7.1. Feature Space Selection and Classifier Training

As mentioned previously, some specific neuroimaging markers (i.e., NDI measures) may be more sensitive for detecting the subtle neurological changes occurring in GWI cases [16]. For training the classifiers, each type of imaging measures (i.e., measurement domains) serves as prior information that will allow us to set up specific feature space for potentially better ML outcomes. Within each feature space, reinforcement learning-based BSO (QBSO) was used to perform iterative search of the subset of features that provides the best classification performance on the training dataset (more details described in QBSO Tuning). Through QBSO, a final subset of features (final solution) was selected to build a final classifier. Final classifiers trained on each feature space were- then tested on the validation dataset (see more details in Ensemble Approach).

##### QBSO Tuning

This feature selection concept combines the BSO and reinforcement learning (specifically Q-learning) to upgrade simple local search to a more adaptive and efficient search for the final solution [34,35]. Previous study has shown that this hybrid method outperforms other well-known ML algorithms for feature selection [35]. More specifically, the BSO method mimics the foraging behavior of natural bees by performing iterative local search for an optimized solution [36].

From the predefined feature space explained earlier, the initial solution is randomly generated. Then, BSO randomly modifies the initial solution to multiple different secondary solutions, where each will be assigned to a bee (an agent) to perform local search to find local optimum (based on k-fold cross-validation accuracy). In this local search stage, each bee refers to a series of experiments obtained in previous steps to make a decision to do further search in the current search pace, and this local search will continue until no further improvement of accuracy occurs. When the bee reaches this point, each bee’s search history is shared to other bees and used for the diversification of searching process.

In the diversification process, the most distant solution will be selected based on the shared information. During this process, the role of reinforcement learning is to allow the agent learn through an interactive environment by trial and error. As the result, the QBSO method will search for a solution (i.e., resulting feature list) that maximizes the reward through multiple iterations. In each iteration, KNN runs on the candidate features (one of the secondary solutions) selected from the bee and tested for 5 iterations of 5-fold cross-validation on the training dataset. We used an average accuracy measure from the 5-fold cross-validation for estimating the reward. Finally, the search process will terminate based on the pre-defined parameters. To set up the optimal parameters, we used a grid-search strategy that is empirically searching the parameters resulting in the highest classification accuracy for the training dataset. The final parameters used in this experiment are listed as follows: flip: 20, max. chance: 9, nBees: 30.

##### Ensemble Approach

Per each feature space (i.e., one type of imaging measure), QBSO produces a subset of final features that provides the highest average accuracy from the iterative search. QBSO is repeated 5 times in total to generate 5 final solution candidates for a single training dataset. Per each solution, we built 3 different classifiers- KNN, support vector machine, and random forest classifiers. The training dataset was further split into 2 parts (i.e., training and testing) and used to train each classifier. Then the weighted majority voting was used to ensemble those 15 classifiers (i.e., 3 classifiers from each solution) to make a final prediction on the validation dataset. The following weight function was used: *Wi* = *Pi*/(1 − *Pi*), *Pi*: performance of *i*-th classifier, *i* = [1:15].

#### 2.7.2. Comparing Classification with Different Imaging Measures

As mentioned previously, each type of imaging measures was used to set up distinct candidate feature space for training the classifiers. The resulting 14 different classifiers (4 NDI, 4 DTI, and 6 T1W-MRI morphometric measures) were evaluated based on their classification performances. For the benchmark testing, the entire dataset was initially divided into a training dataset and a validation dataset based on a 5-fold partitioning. We took one fold as a validation dataset and used the remaining 4-fold data for performing the QBSO training framework (Section 2.7.1). This process was repeated 5 times as training/validation datasets rotate among the 5 folds (by taking each fold as the validation dataset in each iteration). For the classification performance comparison, we reported performance measures (averaged from 5 iterations after validation) of accuracy, sensitivity, specificity, and F-score. We included F-score as a more representative performance measure for the imbalanced case and control groups [37]. In addition to the average accuracy, we included the standard deviation (SD) of accuracy, as an estimate of variations between iterations, and the highest accuracy value for the top three classifiers.

## 3. Results

### 3.1. Group-Level Statistical Comparison and Key Imaging Features

**Figure 1:**
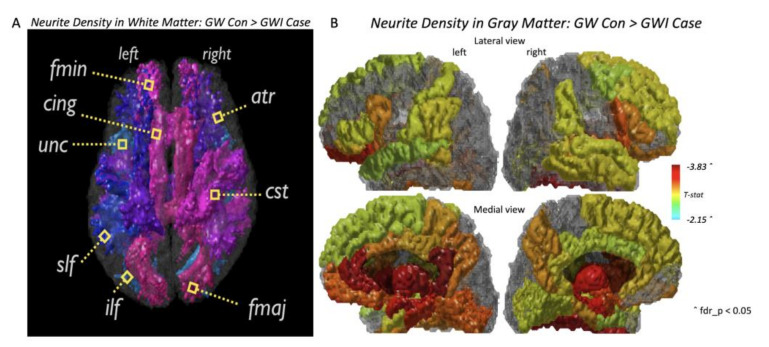
Gulf War illness (GWI) cases vs. Gulf War (GW) control group comparisons of gray matter (GM) and white matter (WM) neurite density imaging (NDI) measures and summary of significant regions. (**A**) 3D tract representation of significant WM ND differences between GWI case and control groups. (**B**) 3D region of interest (ROI) representation of significant GM ND differences between GWI case and control groups. Color bar corresponds to the magnitude of t-value, red indicates greater difference between groups, and vice versa. Fmaj = corpus callosum forceps major, Fmin = corpus callosum forceps minor, atr = anterior thalamic radiations, cst = corticospinal tract, cing = cingulum cingulate gyrus bundle, ilf = inferior longitudinal fasciculus, slf = superior longitudinal fasciculus, unc = uncinate fasciculus.

Statistical analysis of NDI measures showed significant differences between GWI cases and controls in both WM tracts and GM ROIs (FDR-*p* < 0.05) (Figure 1). The full result can be found in Table S1. All major WM tracts showed significant decreases in ND and OD for GWI cases compared to controls (Figure 1A). The greatest significant group differences between GWI cases and controls were seen in the bilateral corticospinal tract (CST, t = −3.119 FDR-*p* = 0.017 (left), t = −3.129, FDR-*p* = 0.017 (right)) and the bilateral anterior thalamic radiations (ATR, t = −2.891, FDR-*p* = 0.017 (left), t = −2.808, FDR-*p* = 0.017 (right)) for WM ND, and in the bilateral cingulum cingulate gyrus bundle (CCG, t = −4.041 FDR-*p* = 0.002 (left), t = −3.384, FDR-*p* = 0.007) for WM OD. Both ND and OD showed decreased patterns (FDR-*p* < 0.05) for most GM ROIs as well (Figure 1B). The greatest significant group differences between GWI cases and controls were seen in the left isthmus of cingulate gyrus (t = −3.319, FDR-*p* = 0.036) and the bilateral thalamus proper (t = −3.168, FDR-*p* = 0.036 (left), t = −3.015, FDR-*p* = 0.036) for GM ND, and in the bilateral caudal anterior cingulate gyrus (t = −3.262, FDR-*p* = 0.016(left), t = −3.182, FDR-*p* = 0.016 (right)), the bilateral posterior cingulate gyrus (t = −3.832, FDR-*p* = 0.016 (left), t = −2.461, FDR-*p* = 0.03 (right)), the bilateral amygdala (t = −3.593, FDR-*p* = 0.016 (left), t = −3.516, FDR-*p* = 0.016 (right)) and the bilateral putamen (t = −3.228, FDR-*p* = 0.016 (left), t = −3.134, FDR-*p* = 0.016 (right)) for GM OD. The full list of statistically significant imaging features can be found in Table S1.

### 3.2. Machine Learning Classification Performance

**Figure 2:**
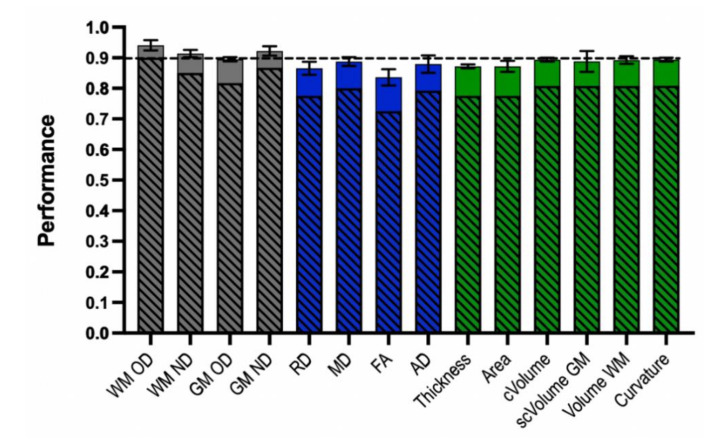
Classification performances of all classifiers. Each bar represents the performance (solid-colored bar: average F-score, shaded area: average accuracy) of each type of classifier trained on one imaging measure, data is presented as mean ± SEM after cross-validation. Grey-colored bars: NDI measure-based classifiers. Blue-colored bars: diffusion tension imaging (DTI) measure-based classifiers. Green-colored bars: T1-weighted structural MRI (T1W-MRI) measure-based classifiers. WM OD = white matter orientation dispersion, WM ND = white matter neurite density, GM OD = grey matter orientation dispersion, GM ND = grey matter neurite density, RD = radial diffusivity, MD = mean diffusivity, FA = fractional anisotropy, AD = axial diffusivity, thickness = cortical thickness, area = cortical surface area, cVolume = cortical volume, scVolume = subcortical GM volume, volume WM = white matter volume.

As shown in Figure 2 and Table 2, the best classifier for GWI cases vs. control we had is trained using the WM OD measures, which achieved F-score of 0.941, an accuracy of 90% (SD: 0.063, highest accuracy: 91.7%), sensitivity of 95%, and specificity of 65%. The specific features include the left CST, the corpus callosum forceps minor (fminor), the left inferior fronto-occipital fasciculus (IFOF), the left inferior longitudinal fasciculus (ILF), the left superior longitudinal fasciculus (SLF), and the left superior longitudinal fasciculus temporal (SLFT). All features were statistically significant based on group-level analysis (Figure 1A, Table S1). The second-best classifier is trained using the GM ND measures, which achieved F-score of 0.922, an accuracy of 86.7% (SD: 0.054, highest accuracy: 91.7%), sensitivity of 96%, and specificity of 40%. The specific features used by this GM ND classifier include both cortical and subcortical structures of the limbic system, including the bilateral caudal anterior cingulate gyri (Table 2). The third best classifier was trained using the WM ND measures, which achieved F-score of 0.914, an accuracy of 85% (SD: 0.048, highest accuracy: 91.7%), sensitivity of 96%, and specificity of 30%. For this classifier, the specific features included the bilateral anterior thalamic radiations (ATR), the bilateral IFOF, the bilateral ILF, the left SLF, the right SLFT and the Fminor (Table 2). The full list of imaging features used by the top three classifiers can be found in Table 2 and the full list of classifier performances can be found in Table S2.

## 4. Discussion

In this study, we used various neuroimaging techniques (NDI, DTI, structural T1W-MRI) to identify important features that may help to differentiate between veterans with GWI and control veterans. These features were selected through two different analytical frameworks: (1) group-level statistical analysis, and (2) single subject-level ML classification models. From our group-level, univariate analysis, we identified important imaging features, especially from WM and GM NDI and T1W-MRI regional volumetric measures, which showed high contrasts between veterans with GWI and control veterans. From the multivariate classification results, we could additionally identify unique imaging features that are important for making single-subject level inferences regardless of its relevance to the group differences.

The results from the group-level statistical analysis showed that NDI measures are the most sensitive marker for detecting GWI pathology than other types of neuroimaging measures. For WM NDI measures, all major tracts showed significant decreases for veterans with GWI compared to control veterans (Figure 1A). The greatest significant group differences were seen in the bilateral CST for WM ND and bilateral CCG bundle for WM OD (Table S1). The roles of these tracts in many essential physical and neuropsychological functions have been well described by previous literatures. For instance, earlier studies showed that disruption of the CST WM integrity was associated with motor impairment that occurs in the early stages of many neurological conditions such as Huntington’s Disease and Multiple Sclerosis [38,39]. Similarly, disruption of CCG has been associated with impaired executive functioning, pain, memory deficits, and has been a main target for conditions including major depression, schizophrenia, post-traumatic stress disorder (PTSD), and autism spectrum disorder [40]. Changes in these tracts captured by our WM NDI results may also be important to understand specific symptoms such as muscle pain, fatigue, and depression observed in GWI.

From the ML framework, we confirmed that WM OD, GM ND, and WM ND measures were the sources of the top three classifiers (based on average accuracy) (Figure 2, Table 2). The classifier trained using the WM OD measure showed the best performance and consistently reporting six features: the left CST, IFOF, ILF, SLF, SLFT, and the Fminor (Table 2). Due to the completely imbalanced distribution of the data used in this study, performance on classifying controls were more challenging in QBSO and this calls better ideas on handling this issue. For example, synthetic oversampling method such as the synthetic minority oversampling technique (SMOTE) may help addressing this issue [41]. Additionally, in this type of imbalanced sample, assessing the F1-score might serve as a more realistic measure of the classification performance [37]. Although we used average accuracy measure for comparing classifiers, WM OD showed a high F-score (0.941), showing that our proposed ML framework is providing reasonable performance at least in this sample. Compared to the NDI classifiers, the classifiers from DTI measures or T1W-MRI measures all had lower classification performance than NDI measures (Table S2). These results suggest that (1) NDI measures are important imaging markers for defining GWI, and (2) the features defined from ML framework provides distinct information from the group-level statistics on describing GWI. While several features from the group-level statistics may present with overlapping patterns to ML classifiers, there are also unique features reported by ML classifiers but not captured in the group-level analysis framework.

Both our findings on group-level statistics and single subject-level classification model demonstrated the importance of NDI measures for defining GWI. Moreover, considering the other ML methods tested on mild or preclinical stage illness, such as mild cognitive impairment staying with ~78% accuracy levels, the classification performance obtained from NDI QBSO is impressive and brings more attention into the complex diffusion imaging measures for studying preclinical stage or mildly progressive illness [42]. In the current study, we not only identified widespread statistically significant NDI features through group-level analysis, but also demonstrated that WM OD measures trained a better classifier compared to other imaging measures. This is consistent with our previous studies on NDI showing that this technique is sensitive to microstructural and macrostructural brain alterations and useful for detecting neurological abnormalities in GW veterans [16]. Our result also corroborated with our previous findings that showed a higher sensitivity for the novel NDI measures compared to the common DTI measures (e.g., FA, MD, etc.). As we suggested before, this might be due to the higher specificity of NDI for detecting changes in different tissue components [16]. We previously found that there is a strong correlation between alterations in GM ND measure and worse self-reported fatigue and sleep symptoms, and with upregulated levels of proinflammatory cytokines TNFRI and TNFRII [16]. However, based on our current findings, GM ND measures provided slightly lower classification performance than WM OD and ND measures in this study. In addition, while classifier trained on WM OD resulted in nearly identical final solutions across five iterations of validation, GM measures resulted in more variabilities in the selected feature solutions. This might be due to the differences in dimensional size between WM and GM feature space. GM measures have more numbers of features (more complexity in the feature space) to be searched out during the QBSO process than WM measures, and thereby requiring more delicate optimization process especially in this not-a-large dataset problem. Although further investigations based on larger dataset is key to address the issue, this may also indicate that WM OD measures can be better markers for simply classifying veterans with GWI from control veterans, while GM ND can be a sensitive marker to specific symptom domains. Our results also support the diagnostic value of these NDI markers for clinical applications.

Altogether, these results suggest that the microstructural changes measured by NDI may be attributed to GM and WM deficits following chronic neuroinflammation. In line with this finding, other studies have shown that chronic neuroinflammation related to GWI symptoms may be a result of both morphological and functional changes that occurred in glial cells. For instance, a study using a rat model of GWI showed that exposure to the chemical agent, diisopropyl fluorophosphate (DFP: a sarin surrogate), was associated with fewer numbers of both mature and dividing oligodendrocytes in the prefrontal cortex, which in turn interrupted the neuron-glial interactions [43]. DFP injection also induced neuroinflammation and neurodegeneration in multiple brain regions, which is associated with impaired contextual fear learning in these rats [44]. Similarly, mice exposed to DFP demonstrated epigenetic changes to genes related to the immune and neuronal systems and altered proportions of myelinating oligodendrocytes in the frontal cortex, which led to disrupted synaptic connectivity and WM alterations in GWI [45]. A recent in-vivo positron emission tomography study corroborated these findings and reported elevated levels of translocator protein (TSPO), a protein upregulated in activated microglia and astrocytes, in veterans with GWI compared to control veterans [46]. This elevation pattern was observed in many areas including the precuneus, prefrontal, primary motor, and somatosensory cortices [46]. Considering this evidence, our current findings further support the importance of novel NDI measures for detecting microstructural changes in the brain following chronic neuroinflammation in GWI.

Besides NDI measures, some T1W-MRI measures also demonstrated good performances for classifying veterans with GWI vs. control veterans. Among classifiers trained using T1W-MRI measures, the cortical volume, subcortical volume, WM volume, and mean curvature models achieved 80.8% accuracy, and highlighted key features in the frontal and temporal regions (Table S2). The results on the group-level statistical analysis also showed reduced volumes of frontal regions among veterans with GWI (Table S1). GM atrophy has been well studied as a hallmark for various neuropsychological disorders. Previous studies showed that reduced total cortical and regional frontal lobe volumes are associated with poor subjective sleep quality and increased self-reported frequency of hearing chemical alarm among GW veterans [12,47].

For DTI measures, the best performance was demonstrated by the MD classifier with an accuracy of 80% and F-score of 0.887 (Table S2). There is evidence that DTI measures may correlate with GWI symptom severity. An early study on GWI veterans showed that fatigue, pain, and hyperalgesia are associated with increased AD in the right IFOF [15]. Another study showed that changes in frontal-limbic WM connectivity, as indicated by reduced MD and increased FA in the right cingulate bundle, was associated with higher PTSD symptom severity score among a sample of 20 GW veterans [48]. In addition, GW veterans who had been exposed to chemical agents have increased AD throughout many regions of the brain including the temporal stem, cingulum bundle, IFOF, etc., compared to unexposed veterans [13]. Through our results, we found that while T1W-MRI and DTI measures are less significant based on group-level statistical analysis, a subset of the regional measures may still explain key components of GWI symptoms.

In this study, we showed that neuroimaging markers help to identify GWI Nevertheless, we are expecting that the current approach can be improved in several aspects. One of the limitations of the current work is the imbalanced sample size, where the number of case subjects greatly exceeded the control subjects for building the classification model. This issue is reflected by the higher sensitivity and lower specificity for all the classifiers. To better handle this issue, we are planning to employ an oversampling method on the minority group to balance the samples. In our follow up work, we will also expand our analysis to a larger GW cohort including more control veterans recruited from other sites. Another important future direction is to test if the combination of multiple imaging measures, or combination of imaging and clinical measures (e.g., cognitive scores, inflammatory profiles, etc.) can improve the classification performance. This multivariate approach will be useful for identifying important features from large datasets. In conclusion, our current work provided the first evidence that novel NDI measures are not only useful for defining GWI based on the conventional group-level statistical comparisons, but also constitute key features for building single-subject level ML models for automated diagnostic classification. The features that are highlighted by our analysis suggest neurological changes underlying GWI pathology and support neuroinflammation as a potential target for therapeutic interventions.

## Figures and Tables

**Table 1:** Subject Characteristics.

BU Subjects	GW Control	GWI Case
N	21	98
Age (years)	54.06	52.46
Gender (F/M)	3/18	20/78

**Table 2:** Summary of classification performances and feature characteristics.

Measure	ACC	SEN	SPE	F-Score	Key Features	
**WM OD**	90%	95%	65%	0.941	L CST **	
					L IFOF **	
					L ILF **	
					L SLF **	
					L SLFT **	
					Fminor **	
**GM ND**	86.7%	96%	40%	0.922	L caudal anterior cingulate * L cuneus L inferior temporal L paracentral * L posterior cingulate * L thalamus proper *	R caudal anterior cingulat R lingual R pars orbitalis R amygdala * R putamen *
**WM ND**	85%	96%	30%	0.914	L ATR * L IFOF * L ILF * L SLF * Fminor *	R ATR * R IFOF * R ILF * R SLFT *

ACC: accuracy, SEN: sensitivity, SPE: specificity, F-score: F1 score, WM OD: white matter orientation dispersion index, GM ND: gray matter neurite density index, WM ND: white matter neurite density index, L: left hemisphere, R: right hemisphere, CST: corticospinal tract, IFOF: inferior fronto-occipital fasciculus, ILF: inferior longitudinal fasciculus, SLF: superior longitudinal fasciculus, SLFT: superior longitudinal fasciculus temporal, Fminor: corpus callosum forceps minor, ATR: anterior thalamic radiation. *: FDR-*p* < 0.05 in group-level statistical comparison. **: FDR-*p* < 0.01 in group-level statistical comparison.

## Supplementary Materials

The following are available online at https://www.mdpi.com/2076-3425/10/11/884/s1, Table S1: List of key imaging features based on group-level statistical comparison, Table S2: The classification performance for all classifiers.
