# Supplementary material for: Neuroimaging Markers for Studying Gulf-War Illness: Single-Subject Level Analytical Method Based on Machine Learning
Source: Brain Sci. 2020 Nov 20;10(11):884. doi: 10.3390/brainsci10110884 (PMC7699718; doi:10.3390/brainsci10110884)
Supplement: Supplementary file 1 [file brainsci-10-00884-s001.pdf]

Table S1. List of key imaging features based on group-level statistical comparison.

Measure	WM tract/GM ROI (statistical significance: FDR-p, t-value)	
**cVolume**	L superior frontal (0.048, -3.255)*	
	L pars orbitalis (0.048, -3.060)*	
	L caudal anterior cingulate (0.048, -3.022)*	
	R insula (0.048, -3.125)*	
**WM ND**	L ATR (0.017, -2.891)*	R ATR (0.017, -2.808)*
	L CAB (0.023, -2.352)*	R CAB (0.019, -2.488)*
	L CCG (0.028, -2.239)*	R CST (0.017, -3.042)*
	L CST (0.017, -3.176)*	R IFOF (0.017, -2.722)*
	L IFOF (0.018, -2.651)*	R ILF (0.019, -2.529)*
	L ILF (0.019, -2.474)*	R SLF (0.017, -2.752)*
	L SLF (0.019, -2.577)*	R SLFT (0.017, -2.783)*
	L SLFT (0.019, -2.540)*	R UNC (0.019, -2.434)*
	L UNC (0.019, -2.507)*	Fminor (0.017, -2.788)*
	Fmajor (0.017, -2.805)*	
**WM OD**	L ATR (0.007, -3.009)**	R ATR (0.011, -2.702)*
	L CAB (0.007, -2.986)**	R CAB (0.007, -3.150)**
	L CCG (0.002, -4.041)**	R CCG (0.007, -3.384)**
	L CST (0.007, -3.014)**	R CST (0.021, -2.351)*
	L IFOF (0.007, -3.133)**	R IFOF (0.010, -2.787)*
	L ILF (0.015, -2.511)*	R ILF (0.022, -2.324)*
	L SLF (0.007, -2.993)**	R SLF (0.007, -3.093)**
	L UNC (0.008, -2.871)**	R SLFT (0.011, -2.725)*
	L SLFT (0.008, -2.911)**	R UNC (0.014, -2.549)*
	Fmajor (0.014, -2.588)*	Fminor (0.007, -3.058)**
**GM ND**	L caudal anterior cingulate (0.045, -2.543)*	L amygdala (0.039, -2.861)*
	L fusiform (0.045, -2.525)*	L hippocampus (0.036, -3.012)*
	L isthmus cingulate (0.036, -3.319)*	L putamen (0.036, -3.081)*
	L lateral orbitofrontal (0.039, -2.842)*	L thalamus proper (0.036, -3.168)*
	L lingual (0.041, -2.643)*	R banks of the superior temporal sulcus (0.048, -2.407)*
	L medial orbitofrontal (0.041, -2.638)*	R fusiform (0.036, -3.171)*
	L parahippocampal (0.039, -2.827)*	R lateral orbitofrontal (0.045, -2.499)*
	L paracentral (0.048, -2.402)*	R pars opercularis (0.041, -2.671)*
	L pars opercularis (0.046, -2.476)*	R pars triangularis (0.045, -2.517)*
	L pericalcarine (0.041, -2.670)*	R precuneus (0.048, -2.448)*
	L posterior cingulate (0.048, -2.378)*	R insula (0.041, -2.664)*
	L precuneus (0.045, -2.553)*	R amygdala (0.041, -2.717)*
	L rostral anterior cingulate (0.036, -3.166)*	R hippocampus (0.036, -2.964)*
	L transverse temporal (0.048, -2.392)*	R putamen (0.041, -2.769)*
	L insula (0.041, -2.619)*	R thalamus proper (0.036, -3.015)*
**GM OD**	L caudal anterior cingulate (0.016, -3.262)*	R banks of the superior temporal sulcus (0.030, -2.502)*
	L caudal middle frontal (0.047, -2.176)*	R caudal anterior cingulate (0.016, -3.182)*
	L entorhinal (0.020, -2.839)*	R caudal middle frontal (0.050, -2.150)*
	L fusiform (0.027, -2.570)*	R entorhinal (0.019, -2.941)*
	L isthmus cingulate (0.017, -3.041)*	R fusiform (0.016, -3.117)*
	L lateral orbitofrontal (0.038, -2.340)*	R lateral orbitofrontal (0.025, -2.645)*
	L lingual (0.030, -2.461)*	R medial orbitofrontal (0.037, -2.364)*
	L medial orbitofrontal (0.023, -2.778)*	R middle temporal (0.025, -2.666)*
	L middle temporal (0.046, -2.225)*	R parahippocampal (0.028, -2.531)*
	L paracentral (0.017, -3.025)*	R pars opercularis (0.046, -2.198)*
	L pars opercularis (0.046, -2.209)*	R pars orbitalis (0.025, -2.684)*
	L pars orbitalis (0.046, -2.211)*	R pars triangularis (0.025, -2.625)*
	L pars triangularis (0.030, -2.469)*	R postcentral (0.027, -2.556)*
	L posterior cingulate (0.016, -3.832)*	R posterior cingulate (0.030, -2.461)*
	L precentral (0.020, -2.849)*	R precentral (0.024, -2.713)*
	L precuneus (0.023, -2.743)*	R rostral anterior cingulate (0.017, -3.007)*
	L rostral anterior cingulate (0.020, -2.875)*	R rostral middle frontal (0.043, -2.259)*
	L rostral middle frontal (0.020, -2.862)*	R superior frontal (0.019, -2.921)*
	L superior frontal (0.016, -3.325)*	R superior temporal (0.017, -3.078)*
	L superior temporal (0.039, -2.317)*	R supra marginal (0.031, -2.440)*
	L temporal pole (0.023, -2.764)*	R temporal pole (0.042, -2.275)*
	L insula (0.030, -2.459)*	R transverse temporal (0.038, -2.332)*
	L amygdala (0.016, -3.593)*	R insula (0.025, -2.657)*
	L hippocampus (0.025, -2.637)*	R amygdala (0.016, -3.516)*
	L putamen (0.016, -3.228)*	R hippocampus (0.025, -2.613)*
	L thalamus proper (0.016, -3.282)*	R putamen (0.016, -3.134)*
		R thalamus proper (0.016, -3.150)*

WM: white matter, GM: gray matter, ND: neurite density index, OD: orientation dispersion index, cVolume: cortical volume, ATR: anterior thalamic radiations, CAB: cingulum angular bundle, CCG: cingulum cingulate gyrus bundle, CST: corticospinal tract, Fmajor: corpus callosum forceps major, Fminor: corpus callosum forceps minor, IFOF: inferior fronto-occipital fasciculus, ILF: inferior longitudinal fasciculus, SLF: superior longitudinal fasciculus, SLFT: superior longitudinal fasciculus temporal, UNC: uncinate fasciculus.

*: FDR-p<0.05

**: FDR-p<0.01

Table S2. The classification performance for all classifiers.

Measure	ACC	SEN	SPE	F-score
**WM OD**	90%	95%	65%	0.941
**WM ND**	85%	96%	30%	0.914
**GM OD**	82.7%	94%	20%	0.895
**GM ND**	86.7%	96%	40%	0.922
**RD**	77.5%	89%	20%	0.866
**MD**	80%	95%	5%	0.887
**FA**	72.5%	86%	5%	0.836

**AD**	79.2%	93%	10%	0.879
**Thickness**	77.5%	92%	5%	0.872
**Area**	77.5%	93%	0%	0.872
**cVolume**	80.8%	97%	0%	0.894
**scVolume**	80.8%	92%	25%	0.888
**Volume WM**	80.8%	96%	5%	0.893
**Curvature**	80.8%	97%	0%	0.894

ACC: accuracy, SEN: sensitivity, SPE: specificity, F-score: F1 score, accuracy, L: left hemisphere; R: right hemisphere. cVolume: cortical volume, WM: white matter, GM: gray matter, ND: neurite density index, OD: orientation dispersion index, RD: radial diffusivity, MD: mean diffusivity, FA: fractional anisotropy, AD: axial diffusivity, Thickness: cortical thickness, Area: cortical surface area, cVolume: cortical volume, scVolume: subcortical volume, Curvature: mean curvature.
